# Antidiabetic and Antihyperlipidemic Effects of *Clitocybe nuda* on Glucose Transporter 4 and AMP-Activated Protein Kinase Phosphorylation in High-Fat-Fed Mice

**DOI:** 10.1155/2014/981046

**Published:** 2014-01-16

**Authors:** Mei-Hsing Chen, Cheng-Hsiu Lin, Chun-Ching Shih

**Affiliations:** ^1^Plant Pathology Division, Taiwan Agricultural Research Institute, Council of Agriculture, Executive Yuan, Wufeng District, Taichung City 41362, Taiwan; ^2^Department of Internal Medicine, Fongyuan Hospital, Ministry of Health and Welfare, Fengyuan District, Taichung City 42055, Taiwan; ^3^Graduate Institute of Pharmaceutical Science and Technology, College of Health Science, Central Taiwan University of Science and Technology, No. 666 Buzih Road, Beitun District, Taichung City 40601, Taiwan

## Abstract

The objective of this study was to evaluate the antihyperlipidemic and antihyperglycemic effects and mechanism of the extract of *Clitocybe nuda* (CNE), in high-fat- (HF-) fed mice. C57BL/6J was randomly divided into two groups: the control (CON) group was fed with a low-fat diet, whereas the experimental group was fed with a HF diet for 8 weeks. Then, the HF group was subdivided into five groups and was given orally CNE (including C1: 0.2, C2: 0.5, and C3: 1.0 g/kg/day extracts) or rosiglitazone (Rosi) or vehicle for 4 weeks. CNE effectively prevented HF-diet-induced increases in the levels of blood glucose, triglyceride, insulin (*P* < 0.001, *P* < 0.01, *P* < 0.05, resp.) and attenuated insulin resistance. By treatment with CNE, body weight gain, weights of white adipose tissue (WAT) and hepatic triacylglycerol content were reduced; moreover, adipocytes in the visceral depots showed a reduction in size. By treatment with CNE, the protein contents of glucose transporter 4 (GLUT4) were significantly increased in C3-treated group in the skeletal muscle. Furthermore, CNE reduces the hepatic expression of glucose-6-phosphatase (G6Pase) and glucose production. CNE significantly increases protein contents of phospho-AMP-activated protein kinase (AMPK) in the skeletal muscle and adipose and liver tissues. Therefore, it is possible that the activation of AMPK by CNE leads to diminished gluconeogenesis in the liver and enhanced glucose uptake in skeletal muscle. It is shown that CNE exhibits hypolipidemic effect in HF-fed mice by increasing ATGL expression, which is known to help triglyceride to hydrolyze. Moreover, antidiabetic properties of CNE occurred as a result of decreased hepatic glucose production via G6Pase downregulation and improved insulin sensitization. Thus, amelioration of diabetic and dyslipidemic states by CNE in HF-fed mice occurred by regulation of GLUT4, G6Pase, ATGL, and AMPK phosphorylation.

## 1. Introduction

The prevalence of diabetes mellitus (DM) represents a significant and growing global health problem. Type 2 diabetes mellitus (T2D) accounts for 90% to 95% of all patients [1]. Diabetes mellitus is characterized by hyperglycemia that involves abnormalities in either insulin secretion or action at peripheral tissues, resulting in reducing insulin sensitivity at skeletal muscle and adipose and liver tissues, which represents insulin resistance. Both genetic (heredity) and environmental factors (obesity and leisure life style) play an important role in T2D.

*Clitocybe nuda* (Fr.) Bigelow and Smith (*Lepista nuda, *commonly known as wood blewit or blue stalk mushroom) is an edible woodland mushroom found in Europe, North America, Asia, and Australia [2]. Due to its special fragrance and delicate texture, it has been cultivated in France, Holland, Britain, and Taiwan. Several bioactive extracts from *C. nuda* have been found to exhibit antioxidant and antimicrobial properties [3–6]. The ethanol extracts of *C. nuda* exerted antioxidant activity by flavonoid amount being 8.21 *μ*g mg^−1^ quercetin equivalent, while the phenolic compound amount was 48.01 *μ*g mg^−1^ pyrocatechol equivalent [5]. Flavonoids have been proven to display a wide range of pharmacological actions such as antimicrobial and antithrombotic activities [7]. In food systems flavonoids can act as free radical scavengers and terminate the radical chain reactions that occur during the oxidation of triglyceride [5]. It was reported that the antioxidant activity of plant materials was well correlated with the content of their phenolic compounds [8].

A new drimane sesquiterpenoid including 3-keto-drimenol, 3beta-hydroxydrimenol, and 3beta, 11, 12-trihydroxydrimene had been shown to exert inhibitory activities against two isozymes of 11 beta-hydroxysteroid dehydrogenases (11*β*-HSD1), which catalyze the interconversion of active cortisol and inactive cortisone [9]. Inhibitors of 11*β*-HSD1 are known to have a potential treatment for the metabolic syndrome [10].

Insulin resistance is known to be associated with reduced glucose uptake and utilization by skeletal muscle and adipose tissues and is related to reduced glucose transporter 4 (GLUT4) gene expressions [11, 12]. Impairment of GLUT4 expression, GLUT4 translocation, and/or insulin signaling may affect insulin-stimulated glucose uptake, and that would result in insulin resistance and hyperglycemia [13, 14]. Therefore, the improvements of GLUT4 contents and/or translocation to the plasma membrane have long been regarded as a potential target in the treatment of diabetes mellitus.

The identification of AMP-activated protein kinase (AMPK) phosphorylation as a likely mechanism is particularly interesting in relation to diabetes and obesity because activation of AMPK inhibits lipid synthesis and can improve insulin action [15, 16]. Because AMPK regulates a variety of different metabolic disorders, it is widely recognized as a useful and safe target for the treatment of metabolic disorders such as T2D and dyslipidemia [15, 16].

Many oral agents for the treatment of DM cause adverse effects. As a result, attempts have been made to discover new antidiabetic regiments derived from plants. Many traditional plant treatments for diabetes are used throughout the world, which are frequently considered to be less toxic and result in fewer side effects than synthetic compounds. Anthocyanins (cyanidin or cyanidin 3-glucoside) were reported to have the potency of a unique therapeutic advantage for prevention obesity and diabetes [17]. *C. nuda* is reported to inhibit 11*β*-HSD1 [9]. Inhibition of 11*β*-HSD1 could improve insulin resistance [10]. Based on the fact that the gills of *C. nuda* are of intense bluish-purple color, the total phenolic content on the antioxidant activity of this mushroom extract [8] and the sesquiterpenoid from *C. nuda* could inhibit 11*β*-HSD1; we hypothesized that extract of *Clitocybe nuda* (CNE) could ameliorate insulin resistance. The present study was to investigate the effects of the CNE-mediated glucose and lipid lowering in a diabetic and dyslipidemic mice model. High-fat (HF) diet could induce C57BL6J mice to hyperglycemia, hyperlipidemia, and hypercholesterolemia [18]. AMPK is considered as a therapeutic target for treatment of diabetes and dyslipidemia [15, 16]. Since activation of the AMPK results in increased lipid and glucose catabolism [19] and GLUT4 is involved in glucose transport, the effect of CNE on AMPK activity and GLUT4 is investigated in mice fed with a HF diet. Phosphorylation of Thr 172 of *α* subunits is essential for AMPK activity [20]. As one of the possible mechanisms of action, this study also examined its effect on the expression of genes involved in gluconeogenesis, lipogenesis, and triglyceride lipase in the liver tissue.

## 2. Materials and Methods

### 2.1. Materials and Preparation of Extract of *Clitocybe nuda* (CNE)

The mushroom (or fungal) strain of *Clitocybe nuda* (Tainung stain no. 1) was cultured under compost extract agar medium. The preparation of grain spawn was as follows: wheat grains were washed with distilled water, then boiled for 20 min, and removed from water by filtration. Then, they were added with 1% CaCO_3_, mixed well, then transferred to the flask, and sterilized at 121°C and 1.2 kg/cm^3^ for 1 h. After one day, the hyphal chunk of the described above *C. nuda* was implanted to the flask at 24°C for 15 days for mycelia to cover grains, called grain spawn. The fruiting of *C. nuda* was as follows: the grain spawn of *C. nuda* was mixed with the fermented rice straw compost, incubated at 24°C for 21–28 days for spawn running, and covered with 1-2 cm peat and the condition was 13°C, 90–95% relative humidity, 1000 ppm CO_2_ concentration, and at daylight for 8 h. During this period, they were periodically supplied with water until fruiting, and then the mushrooms were harvested. After being lyophilized, 30 g of the dried mushroom samples was homogenized and extracted with 40 time volumes of hot water under reflux at 100°C for 40 min. The aqueous extract was filtered over Whatman no. 1 paper and the filtrate was evaporated to a small volume. The filtrate was lyophilized, designated the hot-water soluble fraction (CNE), and was stored frozen at −20°C until required. Nutrients contents were as follows: the fruiting of *C. nuda* (per 100 g) contained crude fat, crude ash, and carbohydrate contents are 3.4~5.0 g, 8.0~12.0 g, and 56.0~67.5 g, respectively; mineral contents are 3.8~6.2 g. The total phenolic contents were determined by the Folin-Ciocalteau method [21]. The total phenolic contents of CNE were 1.29%. The polysaccharides of CNE were 12.62% by phenol-sulfuric acid method [22]. The total anthocyanin contents of CNE were 0.045%. The CNE was diluted and adjusted and then was administrated orally to mice in a volume of 0.2, 0.5, and 1.0 g/kg body  weight (C1: 0.2, C2: 0.5, and C3: 1.0 g/kg body  weight), respectively. Distilled water was administered in a similar volume to control mice.

### 2.2. Animals and Experimental Design

All animal procedures were performed as per guidelines provided by the Institutional Animal Care and Use Committee of Central Taiwan University of Science and Technology. The study contained two parts, part 1: oral glucose tolerance test (OGTT). The ICR mice normal mice (*n* = 5) were fasted for 12 h but were allowed to access to 0.2 g/kg, 0.5 g/kg, and 1.0 g/kg extracts of CNE or an equivalent amount of normal vehicle (water) was given orally 30 min before an oral glucose load (1 g/kg body weight). Blood samples were collected from the retroorbital sinus of fasting mice at the time of the glucose administration (0) and every 30 minutes until 3 hours after glucose administration to determine the levels of glucose. Part 2: animal study was as follows: C57BL/6J mice (4-5 weeks old) were purchased from the National Laboratory Animal Breeding and Research Center, National Science Council. Animals were maintained on a 12 h light/dark cycle (light cycle: 7 a.m. to 7 p.m.). Seven days after acclimation, the C57BL/6J mice were divided randomly into two groups. The control (CON) group (*n* = 9) was fed low-fat diet (Diet 12450B, Research Diets, Inc., New Brunswick, NJ, USA), whereas the experimental group (*n* = 45) was fed a 45% high-fat diet (Diet 12451, Research Diets, Inc., New Brunswick, NJ, USA) for 12 weeks. The low-fat diet was composed of protein 20%, carbohydrate 70%, and fat 10%, whereas high-fat diet was composed of protein 20%, carbohydrate 35%, and fat 45% (of total energy, % kcal). After 8-week diet-induction period, the high-fat-treated mice were randomly subdivided into 5 groups (*n* = 9 per group). Extracts of CNE (including 0.2, 0.5, and 1.0 g/kg/day) or rosiglitazone (Rosi; 1% methylcellulose of 10 mg/kg body weight, obtained from GlaxoSmithKline Product number BRL49653 C) were administrated through oral gavage 1 time per day from 9 to 12 weeks of the experiment, and the mice were still on the high-fat diet, while the CON and high-fat control (HF) mice were treated with vehicle only. The body weight was measured weekly throughout the study. The compositions of the experimental diets are shown as described in [23]. At the end of the study, we deprive animal from food (from p.m. 10:00–a.m. 10:00). The next day (the 85th day), the mice were sacrificed for blood and tissue collection and analysis. The mice were untreated with CNE or Rosi at the 85th day. Livers and white adipose tissues (WATs) (including epididymal, mesenteric, and retroperitoneal WATs) were excised according to the defined anatomical landmarks, and the weights of the tissues were measured. Tissues were immediately frozen using liquid nitrogen and then kept at −80°C for the analysis of target gene expression. Heparin (30 units/mL) (Sigma) was added into blood sample. Plasma samples were collected by centrifugation at 1600 ×g for 15 min at 4°C. The separation of the plasma was finished within 30 min. Plasma was obtained for insulin and leptin assay.

### 2.3. Body Weight and Food Intake Assay

Body weight and food intake were monitored. Body weight was measured weekly throughout the study. The pellet food was weighed and followed by placing in the cage food container. After 24 h, the remaining food was weighed, and the difference represented the daily food intake. Unconsumed pellet HF food was discarded each day and fresh pellet high-fat diet was provided to ensure consistent food quality throughout the study. The HF food was stored at 4°C.

### 2.4. Blood Parameters Assay

Blood samples (0.8 mL) were collected from the retro-orbital sinus of fasting mice and the level of glucose was measured by the glucose oxidase method (Model 1500; Sidekick Glucose Analyzer; YSI Incorporated, Yellow Springs, OH, USA). Plasma triglycerides (TG), total cholesterol (TC), and free fatty acids (FFA) were analyzed using commercial assay kits according to the manufacturer's directions (triglycerides-*E* test, cholesterol-*E* test, and FFA-*C* test, Wako Pure Chemical, Osaka, Japan).

### 2.5. Adipocytokine Levels Assay

The levels of insulin and leptin were analyzed by ELISA using a commercial assay kit according to manufacturer's directions (mouse insulin ELISA kit, Shibayagi, Gunma, Japan, and mouse leptin ELISA kit, Morinaga, Yokohama, Japan).

### 2.6. Histopathology of Adipose and Liver Tissue

Small pieces of epididymal WAT and liver tissue were fixed with formalin (200 g/kg) neutral buffered solution and embedded in paraffin. Sections (8 *μ*m) were cut and stained with hematoxylin and eosin. For microscopic examination, a microscope (Leica, DM2500) was used, and the images were taken using a Leica Digital camera (DFC-425-C).

### 2.7. Measurement of Hepatic Lipids

Hepatic lipids were extracted using a previously described protocol [24]. For the hepatic lipid extraction, the 0.375 g liver samples were homogenized with 1 mL distilled water for 5 min. Finally, the dried pellet was resuspended in 0.5 mL ethanol and analyzed using a triglycerides kit as used for serum lipids.

### 2.8. Isolation of RNA and Relative Quantization of mRNA Indicating Gene Expression

Total RNA from the epididymal WAT, skeletal muscle, and liver tissue was isolated with a Trizol Reagent (Molecular Research Center, Inc., Cincinnati, OH, USA) according to the manufacturer's directions. The integrity of the extracted total RNA was examined by 2% agarose gel electrophoresis, and the RNA concentration was determined by the ultraviolet (UV) light absorbency at 260 nm and 280 nm (Spectrophotometer U-2800A, Hitachi). The quality of the RNA was confirmed by ethidium bromide staining of 18S and 28S ribosomal RNA after electrophoresis on 2% agarose gel containing 6% formaldehyde. Total RNA (1 *μ*g) was reverse transcribed to cDNA in a reaction mixture containing buffer, 2.5 mM dNTP (Gibco-BRL, Grand Island, NY, USA), 1 mM of the oligo(dT) primer, 50 mM dithiothreitol, 40 U Rnase inhibitor (Gibco-BRL, Grand Island, NY, USA), and 5 *μ*L Moloney murine leukemia virus reverse transcriptase (Epicentre, Madison, WI, USA) at 37°C for 1 h and then heated at 90°C for 5 min to terminate the reaction. The polymerase chain reaction (PCR) was performed in a final 25 *μ*L containing 1U Blend Taq-Plus (TOYOBO, Japan), 1 *μ*L of the RT first-strand cDNA product, 10 *μ*M of each forward (F) and reverse (R) primer, 75 mM Tris-HCl (pH = 8.3) containing 1 mg/L Tween 20, 2.5 mM dNTP, and 2 mM MgCl_2_. Preliminary experiments were carried out with various cycles to determine the nonsaturating conditions of the PCR amplification for all the genes studied. The primers are shown in Table 1. The products were run on 2% agarose gels and stained with ethidium bromide. The relative density of the band was evaluated using AlphaDigiDoc 1201 software (Alpha Innotech Co., San Leandro, CA, USA). All the measured PCR products were normalized to the amount of cDNA of GAPDH in each sample.

### 2.9. Western Immunoblotting Analysis

Protein extractions and immunoblots for the determination of phospho-AMPK (Thr172) and GLUT4 proteins were carried out on frozen skeletal muscle, liver, and adipose tissue from mice according to a previous report [25]. Briefly, samples (0.1 g) were powdered under liquid nitrogen and homogenized for 20 s in 500 *μ*L buffer containing 20 mM Tris-HCl (pH = 7.4 at 4°C), 2% SDS, 5 mM EDTA, 5 mM EGTA, 1 mM DTT, 100 mM NaF, 2 mM sodium vanadate, 0.5 mM phenylmethylsulfonyl fluoride, 10 *μ*g/mL leupeptin, and 10 *μ*L/mL pepstatin. 40 *μ*g of each homogenate was mixed with an equal amount of 2 × standard SDS sample loading buffer containing 125 mM Tris-HCl (pH = 6.8), 4% SDS, 20% glycerol, 10% *β*-mercaptoethanol, and 0.25% bromophenol blue and boiled for 10 min before electrophoresis.

The protein contents of both phospho-AMPK (Abcam Inc, Cambridge, MA, USA) and GLUT4 (Santa Cruz Biotechnology, CA, USA) were detected by immunoblotting using a rabbit polyclonal antibody. About 0.1 g of liver, muscle, and adipose tissue of mice (*n* = 9) were used for the homogenate samples containing lysis buffer (pH = 6.4) and protease inhibitors. The protein concentration in supernatant was determined with a BCA protein assay kit (Thermo Scientific, Rockford, IL, USA). Twenty micrograms of proteins were separated by electrophoresis on a polyacrylamide gel 10% (SDS-PAGE) and transferred to a nitrocellulose membrane. The membranes were blocked with 5% slim milk in Tris-buffered saline (TBS) (Amersham BioSciences, Uppsala, Sweden) containing 0.05% Tween-20 (Bio Rad, CA, USA) and incubated overnight at 4°C with antiphospho-AMPK and anti-GLUT4 at 1 : 200 dilution. Subsequently, the membranes were washed three times with TBS containing 0.05% Tween-20 and incubated with secondary antibody anti-rabbit (1 : 1000) (JacksonImmuno Research, Laboratories, Inc., PA, USA) for 1 h. Immunoreactive bands were detected with ECL reagent kit (GE Healthcare BioSciences, Buckinghamshire, UK). The density blotting was analyzed using Alpha Easy FC software (Alpha innotech corporation, Randburg, South Africa). Structural proteins GADPH (Santa Cruz Biotechnology, CA, USA) and *β*-actin (Santa Cruz Biotechnology, CA, USA) were obtained by stripping the nitrocellulose membrane proteins of liver, muscle, and adipose tissue, respectively.

### 2.10. Statistical Analysis

Data were expressed as mean ± S.E. values. Whenever possible, data were subjected to analysis of variance, followed by Dunnett's multiple range tests, using SPSS software (SPSS Inc., Chicago, IL, USA). *P* < 0.05 was considered to be statistically significant.

## 3. Results

### 3.1. Oral Glucose Tolerance Test

**Figure 1:**
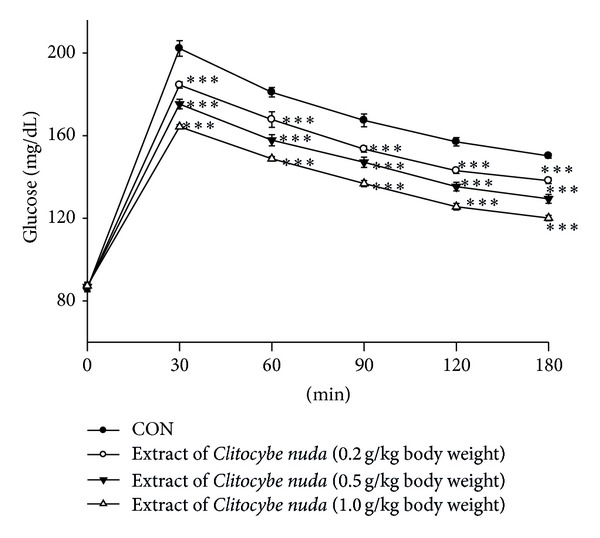
Effects of the extract of *Clitocybe nuda* on oral glucose tolerance in ICR normal mice. Animals in all groups received oral glucose 30 minutes after the extract administration. Blood samples were collected and centrifuged at 3000 rpm for 10 minutes. Each point is the mean ± S.E. of 5 separate mice. ***P < 0.001 was significantly different compared with that of the control group at the same time by ANOVA.

The effect of CNE on OGTT is shown in Figure 1. By the treatment of the ICR mice with 0.2 g/kg, 0.5, and 1.0 g/kg of CNE, the levels of blood glucose were significantly decreased at 30, 60, 90, 120, and 180 min glucose loading when compared with those of the control.

### 3.2. Body Weight, Body Weight Gain, Food Intake and Tissue Weight

**Figure 2:**
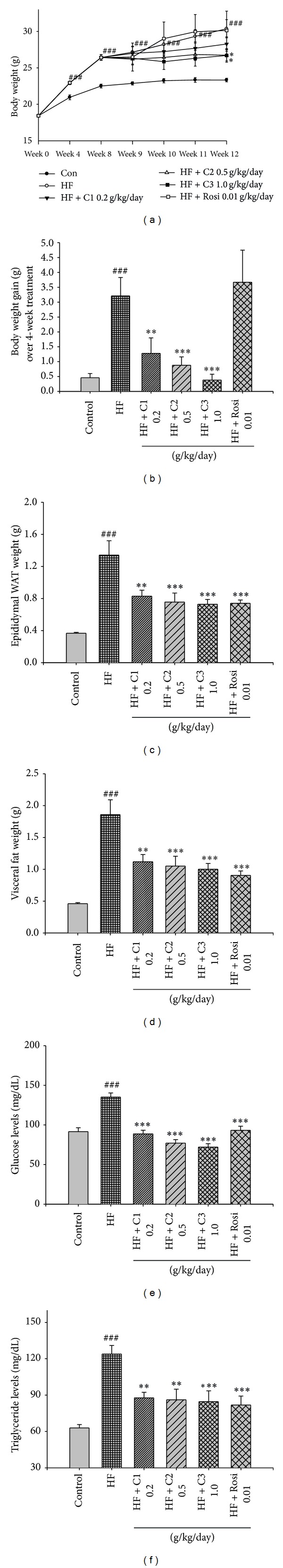
Effects of the extract of *Clitocybe nuda* on (a) body weight at week 12, (b) body weight gain over 4-week treatment, (c) epididymal WAT weights, (d) visceral fat weight, (e) blood glucose levels, and (f) circulating triglyceride levels at week 12. Mice were fed with 45% high-fat diet (HF) or low-fat diet (CON) for 12 weeks. After 8 weeks, the HF mice were treated with vehicle (water; p.o.), extract of *Clitocybe nuda*, or rosiglitazone (p.o.) accompanied with HF diet for 4 weeks. All values are mean ± S.E. (*n* = 9). ^#^ *P* < 0.05, ^##^ *P* < 0.01, and ^###^ *P* < 0.001 were compared with those of the control (CON) group; *P < 0.05, **P < 0.01, and ***P < 0.001 were compared with those of the high-fat + vehicle (distilled water) (HF) group by ANOVA. C1, C2, and C3: extracts of *Clitocybe nuda* (C1: 0.2, C2: 0.5, and C3: 1.0 g/kg body  weight); Rosi: rosiglitazone (0.01 g/kg body  weight). WAT: white adipose tissue; epididymal WAT + retroperitoneal WAT: visceral fat.

All mice groups started with similar mean body weights (17.5 ± 0.1 g). At weeks 8 and 12, the body weight of all the high-fat diet treated mice is significantly greater than that of the CON group (*P* < 0.001, *P* < 0.001, resp.). At week 12, treatment with C2 and C3 showed a significant reduction in body weight compared with that in the HF group (*P* < 0.05, *P* < 0.05, resp.) (Table 2). At week 12, the body weight gain of the HF group is greater than that of the CON group (*P* < 0.001). All the CNE-treated groups showed a significant reduction in body weight gain compared with the HF group (Figure 2(a)). At week 12, the HF group is significantly greater than the CON group in the 4-week cumulative food intake (kcal) (*P* < 0.05). All the CNE-treated groups showed a significant reduction in the 4-week cumulative food intake (kcal) (*P* < 0.001) (Figure 2(b)). At week 12, the weights of absolute adipose tissue (epididymal WAT, visceral fat, mesenteric WAT, and retroperitoneal WAT) were markedly greater in the HF group than those in the CON group (epididymal WAT 265.7%, visceral fat 303.9%, mesenteric WAT 91.6%, and retroperitoneal WAT 454.8.0%) (*P* < 0.001, *P* < 0.001, *P* < 0.001, and *P* < 0.001, resp.) (Table 2 and Figures 2(c) and 2(d)). All the CNE- and Rosi-treated groups showed a significant decrease in the weights of absolute epididymal WAT, visceral fat, mesenteric WAT, and retroperitoneal WAT compared with the HF group. No significant difference in the weights of liver and spleen was observed in all the CNE- and Rosi-treated groups compared with the HF group (Table 2).

### 3.3. Plasma Glucose Levels

At the beginning of the study, all of mice started with similar levels. At weeks 8 and 12, the glucose levels of the HF group were significantly greater than those of the CON group (*P* < 0.001, *P* < 0.001, resp.). Treatment with C1, C2, C3, and Rosi showed a significant reduction in plasma glucose compared with the HF group (*P* < 0.001, *P* < 0.001, *P* < 0.001, and *P* < 0.001, resp.) (Figure 2(e)).

### 3.4. Plasma and Liver Lipid

As time passed, the hypercholesterolaemic phenomenon was evident for the HF diet. As shown in Table 2 and Figure 2(f), at week 12, the levels of TC, TG, and FFA were 62.4%, 97.0%, and 60.8% greater in the HF group than in the CON group (*P* < 0.001, *P* < 0.001, and *P* < 0.05, resp.). All the CNE- and Rosi-treated groups suppressed the HF diet-induced increases in the concentrations of TG. Treatment with C3 suppressed the HF diet-induced increases in the concentrations of TC. Treatment with C2, C3, and Rosi suppressed the high-fat diet-induced increases in the concentrations of FFA. The liver total lipids and triacylglycerol concentrations were 62.8% and 120.2% greater, respectively, in the HF group than those in the CON group (Table 2). Treatment with C1, C2, C3, and Rosi significantly suppressed the HF diet-induced increase in the liver total lipids and triacylglycerol concentrations (Table 2).

### 3.5. Leptin and Insulin Concentrations

As shown in Table 2, at week 12, the concentrations of insulin were greater in the HF group than in the CON group (*P* < 0.05). ALL the CNE-treated groups significantly decreased leptin levels, whereas C2-, C3-, and Rosi-treated groups increased adiponectin levels compared with the HF group. C1-, C2-, C3-, and Rosi-treated groups significantly decreased the levels of insulin compared with the HF group (*P* < 0.05, *P* < 0.01, *P* < 0.01, and *P* < 0.001, resp.).

### 3.6. Histopathology of Adipose and Liver Tissue

**Figure 3:**
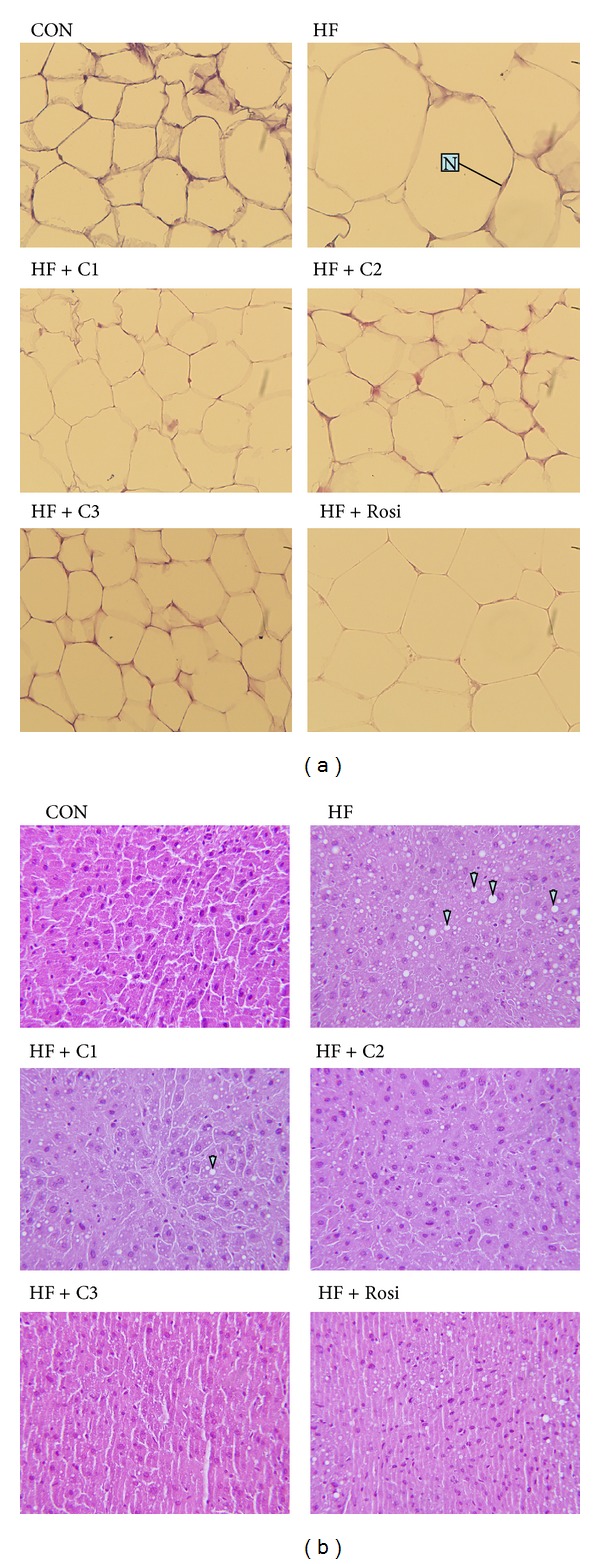
Effects of the extract of *Clitocybe nuda *on liver tissue morphology in the low-fat (Con), high-fat (HF), HF + C1, HF + C2, HF + C3, or HF + Rosi groups. Pictures of hematoxylin and eosin-stained sections of (a) epididymal adipocytes (magnification: 10 (ocular) × 20 (object lens)) from mice fed with extract of *Clitocybe nuda*. White adipose tissue (named adipocytes) is polyhedral by H&E stain, and the appearance showed string-like cytosol surrounding a vacuole. This is because they were embedded in paraffin as immersed in lipid solvents, and finally all the fats were removed. Unobvious nucleus (N) in the other side of cells was carefully observed, and (b) liver tissue (magnification: 10 (ocular) × 40 (object lens)) was obtained from mice fed with extract of *Clitocybe nuda*. The high-fat diet induced the hepatic ballooning degeneration in the HF group as compared with the CON group. The ballooning degeneration is a form of liver parenchymal cell death and the nucleolus was squeezed into the other side named balloon (as the arrow indicated). This may be due to the heap of glycogen in the nucleus. High-fat diet induced obesity and insulin resistance. Insulin levels affected the storage of hepatic glycogen. Treatment with C2 and C3 significantly decreased the degree of ballooning degeneration. Each presented is typical and representative of nine mice. C1, C2, and C3: extracts of *Clitocybe nuda* (C1: 0.2, C2: 0.5, and C3: 1.0 g/kg body  weight); Rosi: rosiglitazone (0.01 g/kg body  weight).

As shown in Figure 3(a), feeding the HF diet induced hypertrophy of the adipocytes compared with the CON group in epididymal WAT. Treatment with C1, C2, and C3 decreased the hypertrophy compared with the HF group. As shown in Figure 3(b), feeding the HF diet induced the ballooning of hepatocyte compared with the CON group in liver tissue. Afterwards, treatment with C2 and C3 decreased the ballooning compared with the HF group. These morphological results strongly suggest that treatment with CNE inhibits the hepatic TG accumulation. The results obtained from the other mice are similar to those shown in Figure 3.

### 3.7. Expressions of ATGL, G6Pase, PPAR*α*, ApoC-III, and SREBP1c in Liver Tissue

**Figure 4:**
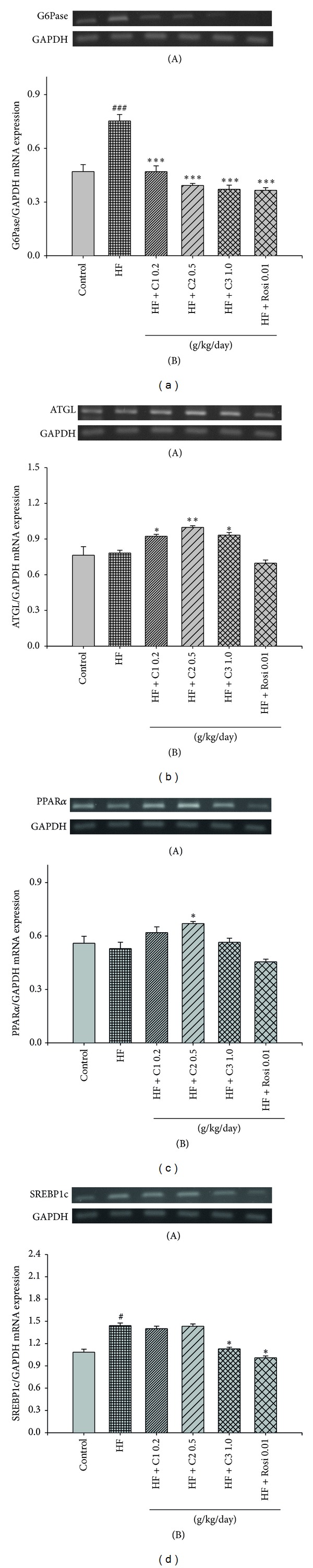
Semiquantative RT-PCR analysis on (a) ATGL, (b) G6Pase, (c) PPAR*α*, and (d) SREBP1c mRNA expressions in liver tissue of the mice by oral gavage extracts of *Clitocybe nuda* (C1: 0.2, C2: 0.5, and C3: 1.0 g/kg body  weight); Rosi: rosiglitazone (0.01 g/kg body weight). Total RNA (1 *μ*g) isolated from tissue was reverse transcripted by MMLV-RT, and 10 *μ*L of RT products was used as template for PCR. The expression levels of ATGL, G6Pase, PPAR*α*, and SREBP1c mRNA were measured and quantified by image analysis. Values were normalized to GAPDH mRNA expression. All values are mean ± S.E. (*n* = 9). ^#^ *P* < 0.05 and ^###^ *P* < 0.001 were compared with those of the control (CON) group; *P < 0.05 and ***P < 0.001 were compared with those of the high-fat + vehicle (distilled water) (HF) group.

As shown Figure 4 and Table 2, at week 12, there were no significant differences in the mRNA levels of ATGL and PPAR*α* between the HF group and the CON group. At week 12, the mRNA levels of G6Pase and SREBP1c were higher in the HF group than in the CON group (*P* < 0.001, *P* < 0.05, resp.). Following treatment, the C1-, C2-, C3-, and Rosi-treated groups significantly decreased the mRNA level of G6Pase (*P* < 0.001, *P* < 0.001, *P* < 0.001, and *P* < 0.001, resp.) (Figure 4(a)). Following treatment, the C1-, C2-, and C3-treated groups increased the mRNA level of ATGL (*P* < 0.05, *P* < 0.01, and *P* < 0.05, resp.) (Figure 4(b)). The C2-treated group increased the mRNA levels of PPAR*α* (Figure 4(c)), whereas the C3- and Rosi-treated groups decreased the apoC-III expression (*P* < 0.05, *P* < 0.01, resp.) (Table 2). The C3- and Rosi-treated groups decreased the SREBP1c expression as compared with the HF group (*P* < 0.05) (Figure 4(d)).

### 3.8. The Phospho-AMPK (Thr172) Protein Contents in Liver Tissue, Skeletal Muscle, and Adipose Tissue

**Figure 5:**
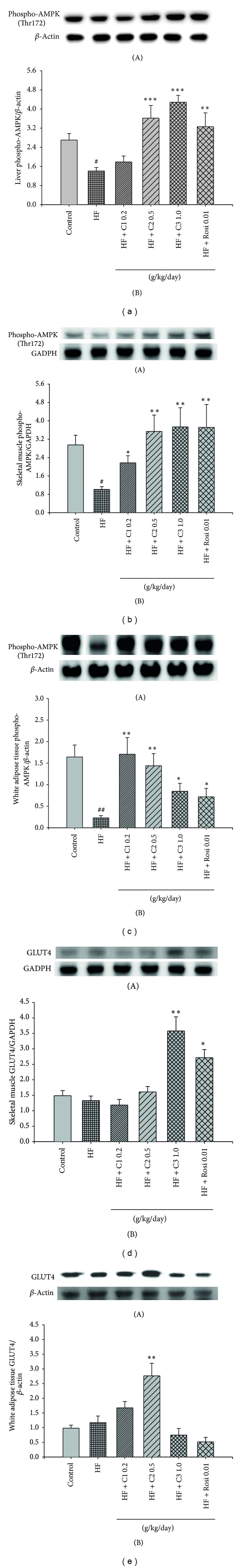
The phospho-AMPK (Thr172) protein contents in (a) liver, (b) skeletal muscle, and (c) white adipose tissue and GLUT4 protein contents in (d) skeletal muscle and (e) adipose tissue of the mice by oral gavage extract of *Clitocybe nuda* for 4 weeks. Protein was separated by 12% SDS-PAGE detected by western blot. All values are mean ± S.E. (*n* = 9). ^#^ *P* < 0.05 and ^##^ *P* < 0.01 were compared with the control (CON) group; *P < 0.05, **P < 0.01, and ***P < 0.001 were compared with the high-fat + vehicle (distilled water) (HF) group. C1, C2, and C3: extract of *Clitocybe nuda* (C1: 0.2, C2: 0.5, and C3: 1.0 g/kg bodyweight); Rosi: rosiglitazone (0.01 g/kg body weight).

At week 12, the protein contents of phospho-AMPK protein were lower in the HF group than those in the CON group in liver, skeletal muscle, and adipose tissue (*P* < 0.05, *P* < 0.05, and *P* < 0.01, resp.). After treatment, the protein contents of hepatic phospho-AMPK were increased in the C2-, C3-, and Rosi-treated groups compared with those in the HF group (*P* < 0.01, *P* < 0.001, and *P* < 0.01, resp.) (Figure 5(a)). Following treatment, the muscular protein contents of phospho-AMPK were increased in the C1-, C2-, C3-, and Rosi-treated groups compared with the HF group (*P* < 0.05, *P* < 0.01, *P* < 0.01, and *P* < 0.01, resp.) (Figure 5(b)). After treatment, the protein contents of adipose phospho-AMPK were increased in the C1-, C2-, C3-, and Rosi-treated groups compared with those in the HF group in liver tissue (*P* < 0.01, *P* < 0.01, *P* < 0.05, and *P* < 0.05, resp.) (Figure 5(c)).

### 3.9. The GLUT4 Protein Contents in Skeletal Muscle and Adipose Tissue

At week 12, there were no significant differences in GLUT4 protein between the HF group and the CON group in skeletal muscle and adipose tissue. After treatment, the skeletal muscular protein contents of GLUT4 were greater in C3- and Rosi-treated groups than those in the HF group (*P* < 0.01, *P* < 0.05, resp.) (Figure 5(d)). Following treatment, the adipose protein contents of GLUT4 were greater in C2-treated groups than those in the HF group (*P* < 0.01) (Figure 5(e)).

## 4. Discussion

*Clitocybe nuda* is an edible woodland mushroom found in Europe; because of its special fragrance and delicacy, it has been cultivated in France and even in Taiwan. The season variation had no effect on the amount of antioxidants property in this mushroom, because the culture is under environmental controls. However, the antidiabetic and antihyperlipidemia activities of* Clitocybe nuda* are not well defined. This study showed that feeding mice with high-fat diet induced hyperglycemia, hyperinsulinemia, hypertriglycemia and hypercholesterolemia. Following treatment of HF-fed mice with CNE, blood glucose, visceral fat mass, and the levels of triglycerides were decreased and improved insulin resistance. This could be because the lowered insulin and glucose levels reflect the glucose output and insulin secretion.

In this study, we found that CNE increased the protein contents of GLUT4 and had favorable effects on glucose uptake into the peripheral tissues and reduced circulating blood glucose. Furthermore, CNE not only increased the phosphorylation of AMPK in tissues, but also improved lipid metabolism. The AMPK activator AICAR has been shown to lower plasma glucose and ameliorate insulin resistance in animal studies [26, 27]. Based on our described findings, we found that CNE is effective in improving insulin resistance and dyslipidemia in a mouse model of type 2 diabetes and dyslipidemia and CNE may have favorable effects on glucose level and lipid metabolism. These findings are involved in the results of GLUT4 contents and AMPK activation.

To investigate the antidiabetic properties of CNE, we chose GLUT4 proteins in skeletal muscle as the major tissues. GLUT4 is a protein that is encoded by the GLUT4 gene found in skeletal muscle. Skeletal muscle is the major tissue responsible for insulin-mediated glucose utilization. Insulin stimulates glucose uptake in skeletal muscle by promoting the translocation of of the GLUT4 to the plasma membrane [28]. Diabetic animals with defective glucose uptake in skeletal muscle appear to be the result of low level of GLUT4 expression [29]. Therefore, the improvements of GLUT4 contents and/or translocation to the plasma membrane have long been regarded as a potential target in the treatment of diabetes mellitus. In the present study there was an increase in the muscular GLUT4 protein contents in the C3-treated HF mice. Increased protein contents of GLUT4 indicated that CNE improved glucose utilization in skeletal muscle by restoring translocation of GLUT4 to the plasma membrane.

The first aim of this study was to investigate the antidiabetic effect and mechanism of CNE. Since the lowering of glucose by CNE and rosiglitazone occurs possibly by different mechanisms, the levels of insulin were measured. Rosiglitazone lowered glucose by insulin sensitizing; therefore, the levels of insulin in the groups of mice treated with Rosi and CNE showed reduction of insulin as a result of insulin utilization. To clarify the mechanism of glucose lowering as well as insulin lowering, mRNA of key transcription factor that included G6Pase gene in the liver was quantitated.

Liver gluconeogenesis is driven by the availability of gluconeogenic substrates and the activity of the important gluconeogenic enzymes like glucose-6-phosphatase (G6Pase) [30]. In the re-feeding state, insulin suppresses gluconeogenesis by transcriptional downregulation of G6pase [31]. Following treatment with CNE, the G6Pase expression was restored to a level lower than the CON group. The hypoglycemic effect is known to be principally attributed to hepatic gluconeogenesis suppression [31], suggesting the lowering of glucose effects of CNE involved in the suppression of hepatic gluconeogenesis.

AMPK activation results in increased glucose uptake in skeletal muscle [32, 33] but decreased hepatic glucose production. AMPK regulates glucose metabolism both via the direct phosphorylation of metabolic enzymes and effects on gene expression [34]. The previous findings suggested that increased AMPK activation is associated with increases in GLUT4 [35]. Once activated, AMPK promotes muscle glucose uptake and inhibits hepatic gluconeogenesis by repressing PEPCK and G6Pase expressions [36]. The present study implicated that CNE exerted hypoglycemic activity and significantly increased the protein contents of phospho-AMPK, whereas expressions of G6Pase were decreased in the liver of all CNE- and rosi-treated mice. Therefore, this might also indicate that CNE by AMPK activation leads to increased GLUT4 contents and decreased G6Pase expression, with stimulation of peripheral glucose uptake and inhibition of hepatic glucose production, thus resulting in the lowering of glucose levels.

GLUT4 translocation is mainly regulated by two independent pathways: the insulin signaling pathway and the AMPK pathway [37]. The kinase, AMPK, has also been shown to regulate GLUT4 translocation [37]. The AMPK pathway is involved in regulation of GLUT4 translocation during exercise or in response to some antidiabetic agents such as AICAR and metformin [37]. In the study, treatment with C2 and C3 was able to increase the phosphorylation of AMPK to a level, suggesting that the AMPK activation is likely responsible for the stimulation of GLUT4 translocation by this CNE. Further studies are needed to shed light on the molecular mechanism involved in the phosphorylation of AS160 pathway.

AMPK activation also has numerous effects on lipid metabolism [38], which leads to the inhibition of fatty acid, triglyceride and sterol biosynthesis in hepatocytes [39, 40], and activation of fatty acid oxidation in skeletal muscle and hepatocytes [41]. In these cases, the effects are known to be due to the direct phosphorylation of metabolic enzyme. Based on a number of studies showing that AMPK regulates a variety of metabolic syndromes, it is widely recognized as a useful target for the treatment of metabolic disorders such as T2D and dyslipidemia [27, 42]. Hence, our findings of activation of AMPK by CNE may implicate this extract as a novel phytonutrient with therapeutic potential for insulin resistant states by targeting AMPK.

The lipid-lowering efficacy of CNE was also caused by upregulation of another enzyme, adipose triglyceride lipase (ATGL), which is responsible for triacylglycerol hydrolase activity in cells that control the rate-limiting step of lipolysis in many insulin sensitive tissues. ATGL exhibits high specificity for triglyceride to hydrolyze into diglyceride and free fatty acid [43]. It is known that activation of AMPK may in turn increase ATGL expression and decrease intracellular lipid droplet accumulation [44]. Recently, ATGL has been considered as a possible therapeutic target for dyslipidemia and fatty liver [45]. In this study, we showed that CNE caused AMPK phosphorylation and increased ATGL expression, which could help triglyceride to hydrolyze, suggesting that CNE could be a possible therapeutic supplementary for dyslipidemia.

In this study, treatment with CNE, the levels of triglycerides were lowered. SREBP-1c plays an important role in response to activation of lipogenic enzyme expression, fatty acid synthesis, and triglyceride accumulation [46]. In PPAR*α*-deficient mice, dysregulation of SREBP-mediated lipogenic genes was noticed [47], suggesting the role of PPAR*α* in SREBP-mediated regulation of lipogenic genes. This study confirmed CNE's lipid-lowering effects partly via downregulation of genes involved in lipid synthesis. Metformin acts by increasing the phosphorylation and activation of AMP kinase, a key enzyme involved in the regulation of gene expression, fuel metabolism, and energy balance [19]. Activation of AMP kinase in liver leads to reduced transcriptional activity of the nuclear factor SREBP1c on the expression of lipogenic enzymes. Therefore, there is a possibility that CNE inactivated these enzymes and/or downregulated gene expression through AMPK activation.

Following treatment with CNE, hepatic PPAR*α* expressions were increased in C2-treated group but leptin levels were decreased in CNE-treated groups. However, we could not exclude the possibility that CNE stimulates PPAR*α* function through leptin-dependent actions. It is known that PPAR*α* is involved in regulation of lipid metabolism and fatty acid oxidation. According to a previous study [48], leptin stimulates fatty acid oxidation through the activation of AMPK and the induction of gene expression, such as PPAR*α*. Thus, further study should shed the light on whether CNE-activated PPAR*α* functions are mediated by leptin-AMPK pathway.

We knew that the gills of *C. nuda* are of intense bluish-purple color. Anthocyanins (cyanidin or cyanidin 3-glucoside) were reported to have the potency of a unique therapeutic advantage for prevention of obesity and diabetes in isolated rat adipocytes [17]. Treatment of adipocytes with anthocyanins enhanced adiponectin secretion and AMPK activation which plays a key role in the metabolic regulation of lipids and glucose uptake in adipocyte [17]. Moreover, dietary cyanidin 3-O-*β*-D-glucoside-rich “purple corn color” (PCC) prevents obesity and ameliorates hyperglycemia in HF-diet-induced mice, and the findings provide a nutritional basis for the use of PCC and anthocyanins as a functional food factor that may have benefits for the prevention of obesity and diabetes [49]. This mushroom extract contained antioxidant properties of the total phenolic content [5]. Our study demonstrated that CNE contained the total phenolic contents. Dietary polyphenols such as resveratrol, catechins, epicatechin, and gallic acid, possess wide therapeutic benefits [50]. Several studies demonstrated the antidiabetic action of polyphenolic class of compounds [51–53]. In the present study, it is possible that the phenolic-rich and anthocyanin contained in CNE may be one of bioactive principles responsible for CNE improved carbohydrate and lipid metabolism in type II diabetic mice. The researcher's interest in polyphenol-rich foods as a dietary source of antioxidants has increased in response to the recognition of the importance of oxidative damage in the pathogenesis of many diseases. Our present investigation provides information that explains, at least in part, the potential benefits of CNE in mice with type II diabetes. However further research is required to establish whether CNE polyphenol in being beneficial for insulin sensitizing and attenuating hyperlipidemic process due to its activity of AMPK activation and glucose uptake by increasing adiponectin levels.

In conclusion, this study demonstrated that CNE increased insulin sensitivity appears to be related to not only increased phosphorylation of AMPK but also increased protein contents of GLUT4 in C3-treated group in skeletal muscle and in C2-treated group in adipose tissue, whereas it is related to a reduction in G6Pase expression, which is one of rate-limiting enzymes of hepatic gluconeogenesis, resulting in reduced glucose level in HF-fed mice. In the other hand, by increasing the phosphorylation of AMPK in liver tissue, CNE should partly decrease hepatic fatty acid synthesis but increase ATGL, which is responsible for hydrolyzing triacyl glycerol, which in turn contributed to the lowering of circulating triglycerides. Therefore, CNE might be a good metabolic regulator and insulin sensitizer. This biological function of CNE might be associated with the increased activity of AMPK. Our findings demonstrated that CNE had the therapeutic potential for the protection against diabetes and hyperlipidemia.

## Figures and Tables

**Table 1:** Primers used in this study.

Gene	Accession numbers	Forward primer and reverse primer	PCR product (bp)	Annealing temperature (°C)
Liver				
ApoC-III	NM_023114.3	F: CAGTTTTATCCCTAGAAGCA R: TCTCACGACTCAATAGCTG	349	47
G6Pase	NM_008061.3	F: GAACAACTAAAGCCTCTGAAAC R: TTGCTCGATACATAAAACACTC	350	50
PPAR*α*	NM_011144	F: ACCTCTGTTCATGTCAGACC R: ATAACCACAGACCAACCAAG	352	55
SREBP1c	NM_011480	F: GGCTGTTGTCTACCATAAGC R: AGGAAGAAACGTGTCAAGAA	219	50
ATGL	AY894805	F: AGG ACA GCT CCA CCA ACA TC R: TGG TTC AGT AGG CCA TTC CT	165	50
GAPDH	NM_031144	F: TGTGTCCGTCGTGGATCTGA R: CCTGCTTCACCACCTTCTTGA	99	55

**Table 2:** Absolute tissue weight, weight gain over 4-week treatment (g), liver lipids, and blood profiles.

Parameter	CON	HF	HF + C1	HF + C2	HF + C3	HF + Rosi
			0.2^a^	0.5^a^	1.0^a^	0.01^a^
Absolute tissue weight (g)						
MWAT	0.310 ± 0.016	0.594 ± 0.044^###^	0.450 ± 0.051**	0.405 ± 0.031***	0.402 ± 0.025***	0.359 ± 0.038***
RWAT	0.093 ± 0.063	0.516 ± 0.068^###^	0.288 ± 0.040**	0.294 ± 0.044**	0.272 ± 0.034***	0.163 ± 0.031***
BAT	0.078 ± 0.005	0.062 ± 0.006	0.065 ± 0.005	0.054 ± 0.006	0.053 ± 0.004	0.071 ± 0.004
Liver (g)	0.873 ± 0.030	0.873 ± 0.029	0.869 ± 0.026	0.841 ± 0.025	0.825 ± 0.030	0.929 ± 0.021
Spleen	0.061 ± 0.004	0.068 ± 0.003	0.078 ± 0.004	0.074 ± 0.002	0.068 ± 0.002	0.074 ± 0.004
Food intake (g/mouse)	76.41 ± 1.57	67.12 ± 1.01^###^	58.97 ± 0.91***	58.71 ± 1.16***	58.71 ± 1.19***	68.11 ± 1.64
Liver lipids						
Total lipid (mg/g)	57.6 ± 2.8	93.8 ± 5.7^###^	75.2 ± 3.9*	65.1 ± 4.5**	63.9 ± 3.9**	64.4 ± 4.9**
Triacylglycerol (*μ*mol/g)	35.6 ± 3.7	78.4 ± 6.9^###^	58.9 ± 6.1*	43.2 ± 4.6***	44.0 ± 5.9***	46.5 ± 5.4***
Blood profiles						
FFA (meq/L)	1.529 ± 0.068	2.472 ± 0.063^#^	1.404 ± 0.192*	1.329 ± 0.161*	1.231 ± 0.156**	1.359 ± 0.173*
TC (mg/dL)	100.3 ± 4.6	154.0 ± 5.5^###^	148.8 ± 3.4	142.6 ± 6.0	130.8 ± 8.8*	134.9 ± 3.5
Leptin (*μ*g/mL)	1.767 ± 0.172	1.958 ± 0.272	1.292 ± 0.092*	1.269 ± 0.121*	1.112 ± 0.173*	1.429 ± 0.161
Insulin (*µ*g/L)	1.567 ± 0.040	1.813 ± 0.101^#^	1.517 ± 0.081*	1.387 ± 0.073**	1.384 ± 0.056**	1.363 ± 0.082***
Adiponectin (ng/mL)	10.05 ± 0.23	8.94 ± 0.49	9.85 ± 0.61	10.57 ± 0.46*	11.21 ± 0.33**	12.19 ± 0.75*
Semiquantitative RT-PCR analysis for mRNA expression in liver tissue						
ApoC-III	1.148 ± 0.039	1.023 ± 0.087	0.990 ± 0.037	0.996 ± 0.063	0.846 ± 0.016*	0.797 ± 0.046**

All values are mean ± S.E. (*n* = 9). ^#^ *P* < 0.05, ^##^ *P* < 0.01, and ^###^ *P* < 0.001 were compared with those of the control (CON) group; *P < 0.05, **P < 0.01, and ***P < 0.001 were compared with those of the high-fat + vehicle (distilled water) (HF) group. C1, C2, and C3: extracts of *Clitocybe nuda.* (C1: 0.2, C2: 0.5, and C3: 1.0 g/kg bodyweight); Rosi: rosiglitazone (0.01 g/kg body weight); BAT: brown adipose tissue; RWAT: retroperitoneal white adipose tissue; MWAT: mesenteric white adipose tissue; FFA: plasma-free fatty acid; TC: total cholesterol. Total RNA (1 *μ*g) isolated from tissue was reverse transcripted by MMLV-RT, 10 *μ*L of RT products was used as template for PCR. Signals were quantitated by image analysis; each value was normalized by GAPDH. ^a^Dose (g/kg/day).

## Abbreviations

AMPK: AMP-activated protein kinase
ATGL: Adipose triglyceride lipase
BAT: Brown adipose tissue
CON: Control
EWAT: Epididymal white adipose tissue
FFA: Free fatty acid
GAPDH: Glyceraldehyde-3-phosphate dehydrogenase
GLUT4: Glucose transporter 4
G6Pase: Glucose-6-phosphatase
HF: High-fat control
HOMA-IR: Homeostasis model assessment for insulin resistance
MWAT: Mesenteric white adipose tissue
PPAR*α*: Peroxisome proliferator-activated receptor *α*
PPARs: Peroxisomal proliferator-activated receptors
Rosi: Rosiglitazone
RT-PCR: Reverse transcription polymerase chain reaction
RWAT: Retroperitoneal white adipose tissue
SREBP-1: Sterol regulatory element binding protein 1
TC: Total cholesterol
TG: triglyceride
WAT: White adipose tissue.
